# Short communication: Five ways UK European Capitals and cities of culture have connected cultural activities with nature and their impacts on health and wellbeing, wider determinants of health and inequality

**DOI:** 10.1016/j.puhip.2024.100533

**Published:** 2024-07-10

**Authors:** Amy Barnes, Kevin Brain, Fiona Phillips

**Affiliations:** a Public Health and Society, Health Sciences, University of York, UK; b Bradford Health Determinants Research Collaboration (HDRC), City of Bradford Metropolitan District Council, UK

**Keywords:** Cultural mega-events, City of culture, Inequality, Determinants of health, Planetary health, Culture-nature initiatives

## Abstract

**Objective:**

To rapidly synthesise evidence for local practice on what initiatives UK European Capitals and Cities of Culture (UKEUCoCs) have implemented connecting cultural activities with green, blue, or outdoor space (culture-nature initiatives) and their impacts on planetary health outcomes: personal health and wellbeing, wider determinants of health particularly the environment, and existing inequality.

**Study design:**

Rapid evidence review.

**Methods:**

A rapid review of published articles and evaluation reports. Published articles were identified through database searches (Proquest, OVID, Scopus, Web of Science, MEDLINE) in January–February 2024. Data was extracted directly into a table and findings synthesised narratively by theme.

**Results:**

Published evidence about UKEUCoC culture-nature initiatives was limited but five initiative types were identified: 1) growing-focused activities; 2) activities exploring human-nature relationships; 3) targeted nature-based wellbeing activities; 4) activities connecting cultural engagement with environmental activism; and 5) use of outdoor spaces for artworks, performances and festivals. UKEUCoC culture-nature initiatives may contribute to short-term improvements in mental health and wellbeing (confidence, self-esteem, subjective wellbeing), community health (community relations, civic pride), cultural participation, and local environmental quality and use, but risk widening existing inequalities. Co-creating initiatives at hyper-local levels with marginalised groups and trusted Community Champions, active involvement, and creating equitable access to livelihood opportunities may mitigate inequality risks.

**Conclusions:**

Evidence is limited but suggests UKEUCoC culture-nature initiatives could positively support planetary health outcomes in the short-term. Equity in these outcomes appears to rely however, on action to ensure the involvement of and sustainable livelihood creation for marginalised groups. It is unclear how outcomes are generated across the initiative types identified, including through interactions between them, where they are implemented. The five initiative types identified in this work could be targeted for further investigation in research and practice on culture-nature initiatives for health more generally, using a complex systems approach to evaluation.

## 1 What this study adds

• First study to identify and develop a typology of culture-nature initiatives (derived from a focused review on UKEUCoCs) and their impacts on planetary health outcomes: personal health and wellbeing, wider determinants of health including environmental quality, and existing inequality.
• Identifies five different culture-nature initiatives: 1) growing-focused activities; 2) activities exploring human relationships to nature; 3) targeted nature-based wellbeing activities; 4) activities connecting cultural engagement with environmental activism; and 5) use of outdoor spaces for artworks, performances and festivals.
• Finds that if culture-nature initiatives within the context of UKEUCoCs are: co-created at a hyper-local level with marginalised groups and trusted Community Champions; involve their active participation; and provide these groups with sustainable livelihoods opportunities, they offer promise in promoting equitable short-term improvements in mental health and wellbeing, community health, cultural participation, and environment quality and use.

## 2 Implications for policy and practice

• Ensure cultural mega events and also future research and practice on culture-nature initiatives more broadly, takes account of the five different initiative types and their potentially differing routes to impact; embedding this in a complex systems approach to their evaluation (with clear theories of change and which take account of potential feedbacks between different initiatives and the role of co-creation and participation in addressing inequity).
• Ensure culture-nature initiatives are: co-created with marginalised groups at hyper-local levels with Community Champions; actively involve people; and provide sustainable livelihood opportunities for marginalised groups (e.g. via routes to education, secure incomes, quality jobs).

## 3 Introduction

Cultural mega-events are attractive to policymakers as they are seen as a route to economic and social regeneration, through attracting additional investment and tourism to an area, reshaping a place's ‘visibility’ nationally and internationally, and fostering a renewed sense of cultural appreciation, community connection and civic pride [[1], [2], [3], [4]]. European Capital of Culture and UK City of Culture programmes are emblematic of cultural mega-events and are competitively awarded by policymakers on a regular basis. In the UK, 5 cities have successfully competed to be a European Capital or City of Culture (Glasgow and Liverpool, as European Capitals of Culture in 1990 and 2008 respectively before the UK left the European Union, and Derry/Londonderry (2013), Hull (2017) and Coventry (2021) as Cities of Culture), with many others having bid to be a host [5].

Despite policy enthusiasm and notwithstanding methodological difficulties in evaluating these events, literature suggests that they have mixed socio-economic outcomes, though findings depend on the success measure used and level of analysis. For example, socio-economic findings are more positive for an area overall, but mixed for different population groups [[6], [7], [8]]. Few studies draw together learning, especially for public health, despite potential impacts on recognised determinants of health and inequality. There are also evaluation gaps in understanding how cultural mega events and different initiatives within them work, for which population groups, and why.

It is in this context that Bradford in West Yorkshire, Northern England, will become UK City of Culture 2025 (BD2025). To support the development of BD2025, [academic-policy-hub-name-anonymised for submission] completed a rapid review for BD2025 stakeholders: the aim (defined and agreed by BD2025 stakeholders) was to quickly synthesise evidence on what initiatives UKEUCoCs have implemented connecting cultural activities with green, blue, or outdoor space (hereafter culture-nature initiatives) and their impacts on personal health and wellbeing, wider determinants of health, and inequality.

Interest in culture-nature initiatives extends beyond UKEUCoCs. There is an increasing breadth of research and practice exploring how to harness the potential of culture-nature initiatives to advance a planetary health agenda: supporting wellbeing and tackling health inequalities, whilst also regenerating the environmental resources on which our wellbeing depends (e.g. through ‘social prescribing’ activities like horticulture and outdoor arts and crafts to achieve health-environment co-benefits) [[9], [10], [11], [12], [13]]. There are gaps in understanding however, about how such initiatives work, for whose health, and with what co-benefits for the environment [12]. While a full review on this topic is needed, we were only able to complete a focused review on culture-nature initiatives within UKEUCoCs due to practice concerns and a tight review timescale, which is why we are sharing our findings as a short communication.

## 4 Methods

A rapid review was completed within limited time (one month) and budget to provide insight for BD2025 stakeholders. Rapid reviews rationalise systematic review methods in order to balance academic rigour with meeting the needs of practice [14]. Relevant Cochrane [14] and Health Policy and Systems guidance [15] on rapid reviews was therefore used to structure the work.

### 4.1 Searches

Database searches (Proquest, OVID, Scopus, Web of Science, MEDLINE) in January–February 2024 identified published articles using simple search terms (see Appendix 1). Article citation and reference tracking was also completed. Evaluation reports were identified by searching key UKEUCoC-related websites. The full strategy is available from the authors.

### 4.2 Inclusion criteria

The review included: published articles of any type and evaluation reports reporting any information related to UKEUCoC initiatives connecting culture-nature and/or their impacts; written in English; publicly-available; and published since the first UK European Capital of Culture (1990).

### 4.3 Screening and selection of reviews

Titles and abstracts were screened by one reviewer against inclusion criteria. As is common in rapid reviews, there was insufficient resource for second checking [15]. Two-stage screening was used, with initial flagging of possible sources for inclusion to identify those for full document review. Evidence excluded at full review was recorded with reasons.

### 4.4 Data extraction and synthesis

Data was extracted directly into a table and findings synthesised narratively by theme: by initiative/implementation insights and reported planetary health outcome areas that reflected key areas of interest of BD2025 stakeholders, namely personal health and wellbeing, wider determinants of health (e.g. community health, nature-environment, cultural participation, economy) and inequality.

### 4.5 Evidence quality assessment

We used Critical Appraisal Skills Programme checklists [16] relevant to each study design and the AACODS checklist [17] for grey literature to consider quality of identified literature (see Appendix 2). Appraisal was not applicable for principally descriptive/reflective studies. Only a small proportion of reported data was relevant in included articles, with a lack of detailed published evidence overall. We also included evaluation reports that were unclear about methods and the underpinning evidence base. We include general reflection on the overall quality of evidence and limitations within our synthesis and discussion.

## 5 Results

**Fig. 1:**
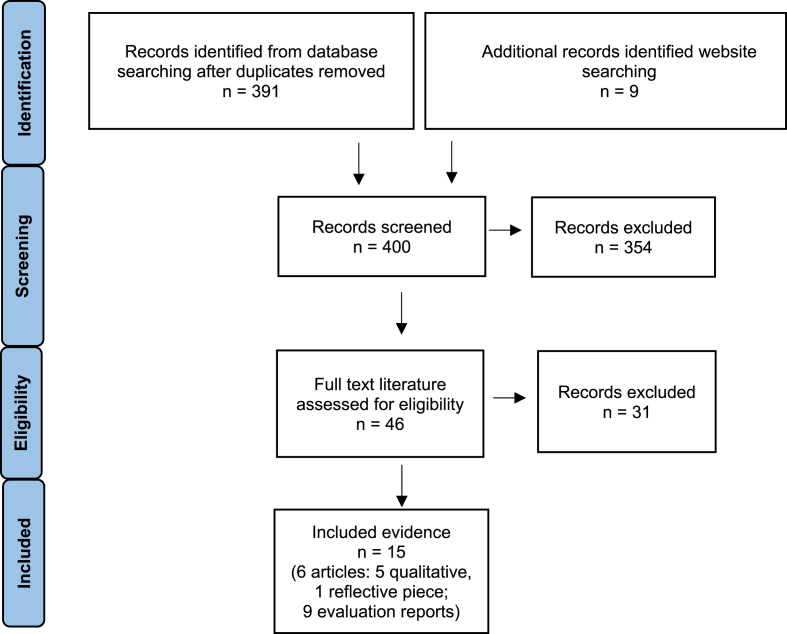
PRISMA diagram indicating process of rapid literature search and selection.

A total of 15 sources were included: 6 articles (1 narrative commentary, 5 qualitative studies) and 9 evaluation reports (Fig. 1); 4 on Hull, 3 on Liverpool, 3 on Coventry, 3 on Derry/Londonderry and 2 more generally on UKEUCoCs. There has been limited focus on culture-nature in previous UKEUCoCs, with Coventry the first to explicitly focus on this in 2021 [[18], [19], [20]]. Consequently, most of the evidence specifically on UKEUCoC culture-nature initiatives related to Coventry and in only 2 evaluation reports, in which methodological information was limited. The other included articles and reports provided more general evidence about UKEUCoCs involving outdoor space which was relevant and so is reported here.

## 6 Synthesis of findings

### 6.1 Culture-nature initiatives in UKEUCoCs

Five different culture-nature initiatives were identified.

1) Growing-focused activities

Coventry City of Culture sought to build interest, knowledge and skills about horticulture, biodiversity, environmentally-friendly growing and community-led food production; for example via city-wide art-pollinator planting and allotment events connecting nature-food-social relationship building [20,21].

2) Targeted nature-based wellbeing activities

Coventry included targeted nature-based wellbeing activities, such as ‘woodland wellbeing’ sessions for people experiencing mental health challenges [20,21].

3) Activities promoting human relationships to nature

Coventry, Hull and Liverpool programmes included activities exploring human relationships to nature. In Coventry, this included guided walks, a young people's Forest camp, habitat co-design work, and multi-sensory co-produced artworks [[20], [21], [22]].

4) Activities connecting cultural engagement with environmental activism and literacy

Coventry organised activities connecting art and culture to environmental activism, to promote a sense of agency in creating a better future (e.g. via citizen science activities and ‘Walking Forest’ events connecting tree loss to women's activism) [20,21].

5) Use of outdoor spaces for artworks, cultural performances and festivals

All UKEUCoCs used green, blue or outdoor space as venues for festivals, displaying artworks and cultural performances, including focusing on nature-related themes to promote learning and/or change how green space was seen and used (e.g. from dividing line to shared space) [6,18,20,23].

We also identified cross-cutting activities that could support the implementation of culture-nature initiatives in cultural mega-events and achievement of positive outcomes from them: 1) creating opportunities for active participation, such as via volunteering outdoors, given the positive wellbeing benefits highlighted in previous UKEUCoCs of this [24,25]; 2) mainstreaming environmentally-responsible planning (e.g. via a Green Code) to reduce the risk of events having negative environmental impacts [20,26];3) embedding community involvement and co-creation in event planning and implementation, including by working with Community Champions (respected local individuals/organisations) to ensure inclusion of marginalised groups in cultural participation and their access to wider event benefits; and 4) ensuring that programming creates equitable routes to education, secure incomes and quality employment [[19], [20], [21],^23^,^25^,^27^].

### 6.2 Outcomes

There was limited insight in included sources about outcomes of the five different culture-nature initiatives identified. Outcomes for culture-nature initiatives tended to either be reported together and/or (particularly in the case of using outdoor space for artworks, performances and festivals) as part of outcomes for UKEUCoCs overall. Outcome measures were also different and/or unclear in included evidence.

#### 6.2.1 Personal health and wellbeing

Culture-nature initiatives may have positive short-term mental health and wellbeing impacts for those involved, with active participation (such as volunteering outdoors at performances and in targeted nature-based wellbeing activities) particularly supporting positive short-term improvements in confidence, self-esteem, subjective wellbeing and mental wellbeing [24,25,27]. 10.13039/100028163Equity in health and wellbeing benefits may be affected by unequal cultural participation across population groups and widening economic inequalities from UKEUCoCs (e.g. via inflation, rising living costs, gentrification) [23,27].

#### 6.2.2 Community health

Included evidence consistently reported short-term improvements in community health (community relations, civic pride) in UKEUCoCs including via culture-nature programming, but also exclusions for marginalised groups [23,27]. Hyper-local, community-driven and participatory culture-nature initiatives may be more likely to improve community relationships and civic pride than other cultural events short-term [21].

#### 6.2.3 Nature-environment

Evaluation of Coventry's nature-culture programming suggests that growing activities can create new urban food growing areas and improve the public realm, and activities promoting human-nature connections may increase environmental asset use [21].

#### 6.2.4 Cultural participation

Insight was gathered through forest camps on barriers to young people's cultural engagement with nature [20,21]. Other evidence indicated exclusion risks for marginalised groups in cultural participation, but that community-centred, participatory arts practices might redress this [19,28].

#### 6.2.5 Economy

Two evaluation reports highlighted that partners in Coventry's culture-nature events gained from increased awareness of their activities (e.g. health social enterprise grew its membership base) [20,21]. Other evidence suggested that UKEUCoCs risk worsening economic inequality due to short-term impacts on local inflation, rising living costs, transport disruption, and public space overcrowding, and longer-term gentrification; with negative impacts potentially mitigated via involvement of marginalised groups and programming that creates equitable routes to education, secure income and quality employment [19,21,23,27].

## 7 Discussion

There is limited, high-quality published evidence about UKEUCoC culture-nature initiatives and their impacts on planetary health outcomes: personal health and wellbeing, wider determinants of health, and existing inequality. Few UKEUCoCs have specifically connected culture to nature. There is however, some evidence on the impacts of initiatives involving outdoor space within UKEUCoCs more generally that is of relevance. This evidence draws attention to the risks of inequality from culture-nature initiatives embedded within UKEUCoCs – a risk likely heightened given the ongoing crisis in living costs across the UK and Europe [29] – and thus to the importance of considering the context within which culture-nature initiatives are implemented to understand their public health impacts.

While our review was limited to focusing on culture-nature initiatives within UKEUCoCs to provide insights for local programming, and because we had to balance the inherent tension between academic rigour in reviews and meeting the specific needs of practice, the findings complement a small but growing wider literature on how to harness culture-nature initiatives to advance planetary health [[10], [11], [12],^30^]. Our review confirms, for example, the potentially valuable role of culture-nature initiatives in contributing to short-term improvements in mental health and wellbeing (confidence, self-esteem, subjective wellbeing), community health (community relations, civic pride), cultural participation, with some (though more uncertain) evidence of changes in environmental quality and use, and draws attention to risks of inequality in ‘who’ experiences health and wellbeing benefits. These findings, while tentative, resonate with a recent review on nature-based interventions that covered 2 of the 5 culture-nature initiative types we identified within UKEUCoCs, namely growing-focused activities and targeted nature-based wellbeing work [12].

Our 5 initiative types expand the understanding of culture-nature initiatives beyond growing and targeted nature-based wellbeing work, to include activities connecting cultural engagement with environmental activism and literacy, and the use of outdoor spaces for artworks, cultural performances and festivals. We suggest that a more detailed review to capture this breadth of culture-nature initiatives for planetary health would be valuable, so as to provide a clearer framework or typology from which to evaluate these in practice, including in contexts of social prescribing. Limitations in the UKEUCoC and wider evidence base mean that it is unclear how and why health and environmental co-benefits might or be produced from culture-nature initiatives [12], and so this kind of evaluative typology could be useful. Future evaluations, including those embedded within UKEUCoCs, will likely require a complex systems approach to evaluation, careful thinking about the role and interaction of different culture-nature initiatives within their implementation contexts, and the development of clear theories of change [12]. It will also require being clear about why hyper-local co-creation with marginalised groups, trusted Community Champions and active participation are important for equity, as well as exploring how to build in sustainable livelihood opportunities within this approach. Power, involvement and income or livelihood security are well-recognised as fundamental determinants of planetary health and inequity [9,31,32] and so drawing on a wider public health literature would be useful in this regard.

## Ethical approval

Ethical approval was not needed as the rapid review did not involve human participants.

## Funding

Barnes is funded by the NIHR Yorkshire and Humber Applied Research Collaboration [reference NIHR200166]) and UK Prevention Research Partnership Collaboration (MRC) - ActEarly [reference MR/S037527/1]. The NIHR Health Determinants Research Collaboration Bradford is funded by the PHR Programme (NIHR151305). Content and views expressed in this paper are those of the author(s) and not necessarily those of the National Institute for Health Research, the Department of Health and Social Care, or the UK Prevention Research Partnership/MRC.

## Declaration of competing interest

Amy Barnes is an academic lead for the University of York/Bradford Health Determinants Research Collaboration (HDRC) Policy Hub – Barnes led the review, analysis and write up.

Kevin Brain and Fiona Phillips work for Bradford Council Health Determinants Research Collaboration (HDRC) and support the joint University of York/Bradford Health Determinants Research Collaboration (HDRC) Policy Hub – Brain and Phillips were involved in agreeing the scope of the work, commented on emerging findings and reviewed/commented on the final manuscript.
