# Supplementary material for: Short communication: Five ways UK European Capitals and cities of culture have connected cultural activities with nature and their impacts on health and wellbeing, wider determinants of health and inequality
Source: Public Health Pract (Oxf). 2024 Jul 10;8:100533. doi: 10.1016/j.puhip.2024.100533 (PMC11301185; doi:10.1016/j.puhip.2024.100533)
Supplement: Multimedia component 1 [file mmc1.docx]

Proquest 24/01/2024

“city of culture”

“capital of culture”

OVID 25/01/2024

All fields – city of culture

Scopus 25/01/2024

Tile, Abstract, Keywords – “city of culture” – limited to journal article

Web of science

“city of culture” - limited to England, Scotland, Northern Ireland and limited to journal article

MEDLINE 25/01/2024

“city of culture” 1

“capital of culture” 3

All exclude

TOTAL through data base searching and removing duplicates = 387 (+4 from MEDLINE search)

TOTAL through database searching for full text review: 36
