# Supplementary material for: Short communication: Five ways UK European Capitals and cities of culture have connected cultural activities with nature and their impacts on health and wellbeing, wider determinants of health and inequality
Source: Public Health Pract (Oxf). 2024 Jul 10;8:100533. doi: 10.1016/j.puhip.2024.100533 (PMC11301185; doi:10.1016/j.puhip.2024.100533)
Supplement: Multimedia component 2 [file mmc2.docx]

**Appendix 2 - Quality appraisal**

For critical appraisal, we used checklists from the Critical Appraisal Skills Programme (Critical Appraisal Skills Programme: <https://casp-uk.net/casp-tools-checklists/>) relevant to each empirical study design, and the AACODS checklist for grey literature, to consider the quality of identified literature. Appraisal was not applicable for predominantly descriptive or theoretical studies.

**Grey literature -** AACODS checklist [modified] 0-5 rating 0=low/poor 5= high/good

| **First author & date** | **Authority** | **Accuracy** | **Coverage** | **Objectivity** | **Date** | **Significance**  **(to specific research area that is a focus of the review)** | **TOTAL** |
| --- | --- | --- | --- | --- | --- | --- | --- |
| Culture, Place and Policy Institute (2018) | 5 | 3 | 3 | 3 | 5 | 2 | 21 |
| Culture, Place and Policy Institute (2021) | 5 | 3 | 3 | 3 | 5 | 2 | 21 |
| Garcia et al (2011) | 5 | 3 | 3 | 3 | 5 | 2 | 21 |
| Ganga et al (2022) | 5 | 4 | 4 | 4 | 5 | 3 | 25 |
| Musella and Bignall-Donnelly (2022) | 5 | 4 | 4 | 3 | 5 | 3 | 24 |
| Melville et al (2010) | 5 | 4 | 3 | 3 | 5 | 3 | 23 |
| Culture Liverpool (2023) | 3 | 1 | 1 | 1 | 5 | 1 | 12 |
| Bek et al (2022) | 5 | 4 | 3 | 4 | 5 | 4 | 25 |
| University of Warwick and University of Coventry (2023) | 5 | 4 | 3 | 4 | 5 | 4 | 25 |

**AUTHORITY**

**Individual author:**

• Associated with a reputable organisation?

• Professional qualifications or considerable experience?

• Produced/published other work (grey/black) in the field?

• Recognised expert, identified in other sources?

• Cited by others? (use Google Scholar as a quick check)

• Higher degree student under “expert” supervision?

**Organisation or group**:

• Is the organisation reputable? (e.g. W.H.O)

• Is the organisation an authority in the field?

**In all cases:**

• Does the item have a detailed reference list or bibliography?

**ACCURACY**

• Does the item have a clearly stated aim or brief?

• Is so, is this met?

• Does it have a stated methodology?

• If so, is it adhered to?

• Has it been peer-reviewed?

• Has it been edited by a reputable authority?

• Supported by authoritative, documented references or credible sources?

• Is it representative of work in the field?

• If No, is it a valid counterbalance?

• Is any data collection explicit and appropriate for the research?

• If item is secondary material (e.g. a policy brief of a technical report) refer to the original. Is it an accurate, unbiased interpretation or analysis?

**COVERAGE**

All items have parameters which define their content coverage. These limits might mean that a work refers to a particular population group, or that it excluded certain types of publication. A report could be designed to answer a particular question, or be based on statistics from a particular survey.

• Are any limits clearly stated?

**OBJECTIVITY**

It is important to identify bias, particularly if it is unstated or unacknowledged.

• Opinion, expert or otherwise, is still opinion: is the author’s standpoint clear?

• Does the work seem to be balanced in presentation?

**DATE**

For the item to inform your research, it needs to have a date that confirms relevance

• Does the item have a clearly stated date related to content? No easily discernible date is a strong concern.

• If no date is given, but can be closely ascertained, is there a valid reason for its absence?

• Check the bibliography: have key contemporary material been included

**SIGNIFICANCE**

This is a value judgment of the item, in the context of the relevant research area

• Is the item meaningful? (this incorporates feasibility, utility and relevance)

• Does it add context?

• Does it enrich or add something unique to the research?

• Does it strengthen or refute a current position?

• Would the research area be lesser without it?

• Is it integral, representative, typical?

• Does it have impact? (in the sense of influencing the work or behaviour of others)

**Quality assessment of qualitative papers**

| **First author, date** |  |  |  |  |  |  |  |  |  |  |
| --- | --- | --- | --- | --- | --- | --- | --- | --- | --- | --- |
| Boland et al (2019) | y | y | y | y | y | n | n | Not clear | y | Provides insights about use of green/outdoor spaces for performances and significance of this for dimensions of public health relevant to review – also more general insights about exclusion of working class communities, and how CoC is managed |
| Devine and Quinn (2019) | y | y | y | y | y | n | n | Not clear | y | Provides insights about use of green/outdoor spaces for performances and significance of this for dimensions of public health relevant to review – in particular in relation to social capital |
| Doak (2020) | y | y | y | Not clear | Not clear | n | n | Not clear | y | Highlights some negative community wellbeing effects of city-centric use of space, including outdoor green-blue space e.g. enhancing feelings of marginality |
| Whelan et al (2023) | Y | y | y | y | y | Not clear | Not clear | y | y | Insights about wellbeing benefits of volunteering, including volunteering in outdoor space/nature |
| Whitfield et al (2023) | y | y | y | y | y | Not clear | Not clear | y | Y | Some insights with regards to volunteering |

For each, Yes, Not clear or No

1. Was there a clear statement of the aims of the research?
2. Was a qualitative methodology appropriate?
3. Was the research design appropriate to address the aims of the research?
4. Was the recruitment strategy appropriate to the aims of the research?
5. Was the data collected in a way that addressed the research issue?
6. Has the relationship between researcher and participants been adequately considered?
7. Have ethical issues been taken into consideration?
8. Was the data analysis sufficiently rigorous?
9. Is there a clear statement of findings?
10. How valuable is the research?
